# Beyond oxygen targets: the DOSE framework as a conceptual model for dynamic oxygen stewardship in retinopathy of prematurity

**DOI:** 10.3389/fped.2026.1882831

**Published:** 2026-09-03

**Authors:** Johanes Edy Siswanto

**Affiliations:** 1 Department of Neonatology, Harapan Kita National Women and Children Health Centre, Jakarta, Indonesia; 2 Faculty of Medicine, Universitas Pelita Harapan, Tangerang, Indonesia

**Keywords:** DOSE framework, dynamic oxygen exposure, low- and middle-income countries, oxygen instability, oxygen saturation, oxygen stewardship, oxygen therapy, retinopathy of prematurity

## Abstract

Retinopathy of prematurity (ROP) remains a major cause of preventable childhood blindness, and oxygen therapy continues to represent a central but complex component of neonatal care. Current oxygen management is largely guided by static peripheral oxygen saturation (SpO_2_) target ranges, commonly around 90%–95%. However, target ranges alone may not fully capture the dynamic nature of oxygen exposure in preterm infants, including fluctuations, intermittent hypoxemia, hyperoxemic excursions, instability, and cumulative exposure over time. We propose the Dynamic Oxygen Stability and Exposure (DOSE) framework as a conceptual and hypothesis-generating model for dynamic oxygen stewardship in ROP prevention. DOSE integrates four interconnected domains: delivered oxygen input (dose), physiologic oxygenation output reflected by the SpO_2_ response, oxygen stability or fluctuation control, and cumulative oxygen exposure over time. The novelty of DOSE does not lie in claiming that these individual elements are new, but in organizing them into a unified framework that may help explain why similar SpO_2_ targets can produce different biologic oxygen experiences across infants and care settings. This framework does not replace existing oxygen saturation guidelines and is not yet a validated clinical score or predictive model. Rather, it provides a structured approach for future research, quality improvement, and prospective validation of dynamic oxygen-exposure metrics. DOSE also recognizes that ROP is multifactorial and that oxygen instability should be interpreted alongside prematurity severity, postnatal growth, inflammation, transfusion, nutrition, respiratory morbidity, screening access, and treatment availability. By shifting attention from static targets alone toward oxygen stability and cumulative exposure, DOSE may support future strategies for safer and more individualized oxygen stewardship in diverse neonatal settings.

## Graphical Abstract

**Figure:**
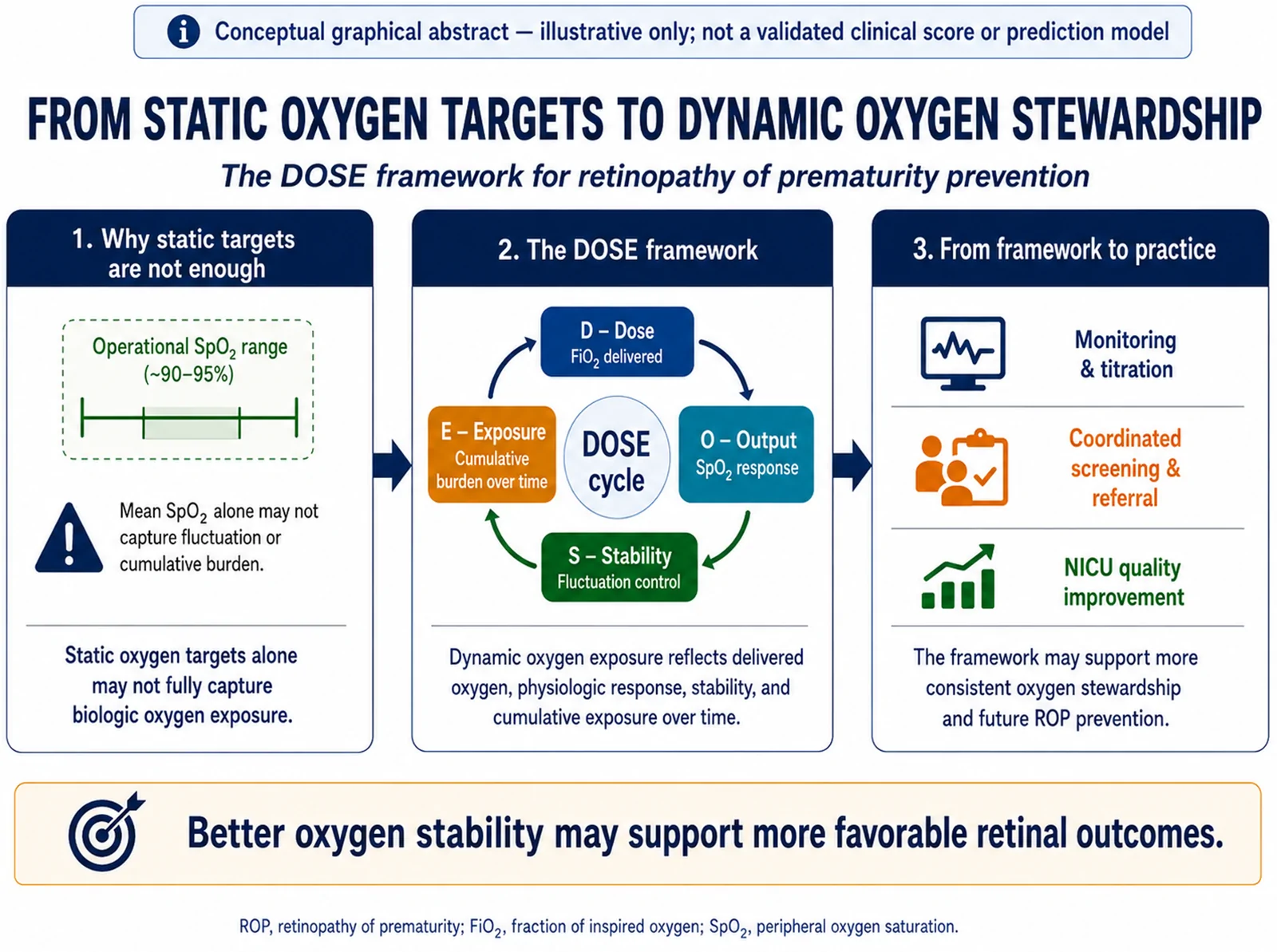
From static oxygen targets to dynamic oxygen stewardship: the DOSE framework**.** The graphical abstract summarizes the conceptual transition from static oxygen targeting toward dynamic oxygen stewardship through the DOSE framework. It presents the rationale for moving beyond mean SpO₂ targets alone, highlights the four DOSE domains, and outlines how the framework may support monitoring, coordinated screening and referral, and NICU quality improvement. It is intended as an illustrative conceptual overview and does not represent a validated clinical score, prediction model, or empirically derived outcome estimate.

## Key message

Oxygen targeting defines where saturation should be, while oxygen stability and cumulative exposure may shape how the immature retina experiences oxygen over time.

From static oxygen targets to dynamic oxygen stewardship: the DOSE framework. The graphical abstract summarizes the conceptual transition from static oxygen targeting toward dynamic oxygen stewardship through the DOSE framework. It presents the rationale for moving beyond mean SpO_2_ targets alone, highlights the four DOSE domains, and outlines how the framework may support monitoring, coordinated screening and referral, and NICU quality improvement. It is intended as an illustrative conceptual overview and does not represent a validated clinical score, prediction model, or empirically derived outcome estimate.

## 1 Introduction

Retinopathy of prematurity (ROP) remains a major cause of preventable childhood blindness worldwide and continues to represent an important challenge in neonatal care, particularly as survival of very preterm and extremely preterm infants improves across diverse healthcare settings (1, 2). Oxygen therapy is central to both neonatal survival and ROP pathophysiology, creating a persistent clinical paradox: insufficient oxygen exposure may increase mortality and systemic morbidity, whereas excessive or unstable oxygen exposure may contribute to oxidative retinal injury and abnormal vascular development (3, 4).

Large oxygen-targeting trials and meta-analyses, including SUPPORT, BOOST II, and the Neonatal Oxygenation Prospective Meta-analysis collaboration, have shaped contemporary neonatal oxygen practice by demonstrating the complex balance between survival, systemic morbidity, and severe ROP (5–7). These studies showed that lower oxygen saturation targets may reduce severe ROP but can be associated with increased mortality, whereas higher targets may improve survival but increase oxygen-related morbidity. As a result, many neonatal guidelines and clinical practices have adopted operational peripheral oxygen saturation (SpO_2_) target ranges around 90%–95% as a pragmatic compromise between competing risks (8).

**Figure 1:**
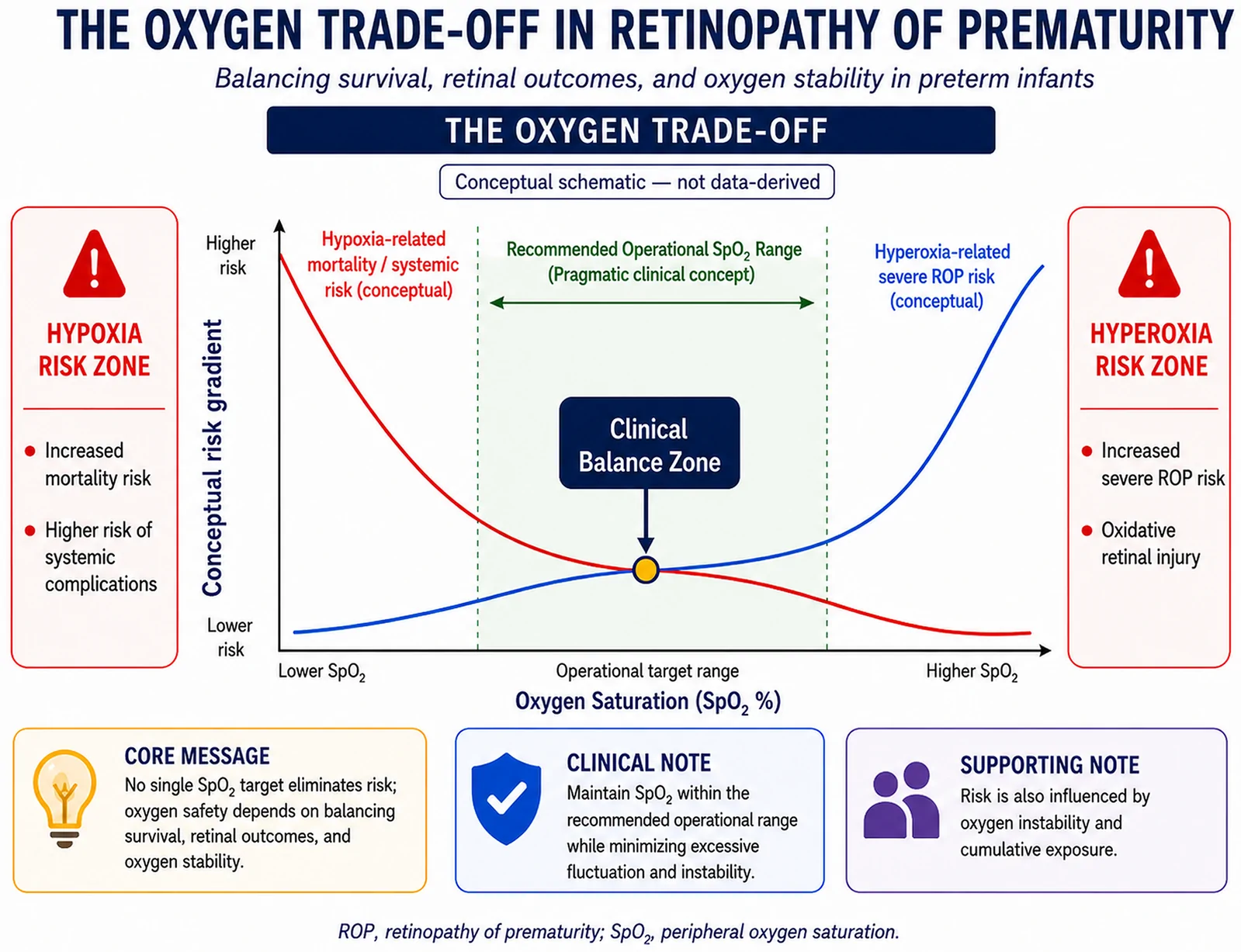
Conceptual illustration of the oxygen trade-off and the limitations of a universal “safe zone” concept. This schematic figure illustrates the theoretical balance between hypoxia-related systemic risk and hyperoxia-related retinal risk in preterm infants. The curves are conceptual and are not derived from empirical risk estimates, patient-level data, or a validated prediction model. The operational SpO_2_ range of 90%–95% is presented as a pragmatic clinical target rather than a universally safe physiologic zone. The figure emphasizes that oxygen safety may depend not only on target-range selection, but also on oxygen stability, fluctuation burden, cumulative exposure, and infant-level vulnerability. The figure is labelled as “Conceptual schematic—not data-derived.”

The conceptual oxygen trade-off and the limitations of a universal “safe zone” are illustrated in Figure 1.

However, static SpO_2_ target ranges alone do not fully capture the dynamic nature of oxygen exposure experienced by preterm infants in real-world neonatal intensive care. Two infants may have similar mean SpO_2_ values yet exhibit markedly different patterns of intermittent hypoxemia, hyperoxemic excursions, fluctuation amplitude, time outside the target range, and cumulative oxygen exposure. These temporal patterns may be biologically relevant because repeated hypoxia–reoxygenation cycles can contribute to oxidative stress, inflammatory signaling, vascular dysregulation, and abnormal retinal angiogenesis (4, 9, 10). Therefore, oxygen-related retinal vulnerability may depend not only on the selected saturation target, but also on the stability, variability, and cumulative burden of oxygen exposure over time.

**Figure 2:**
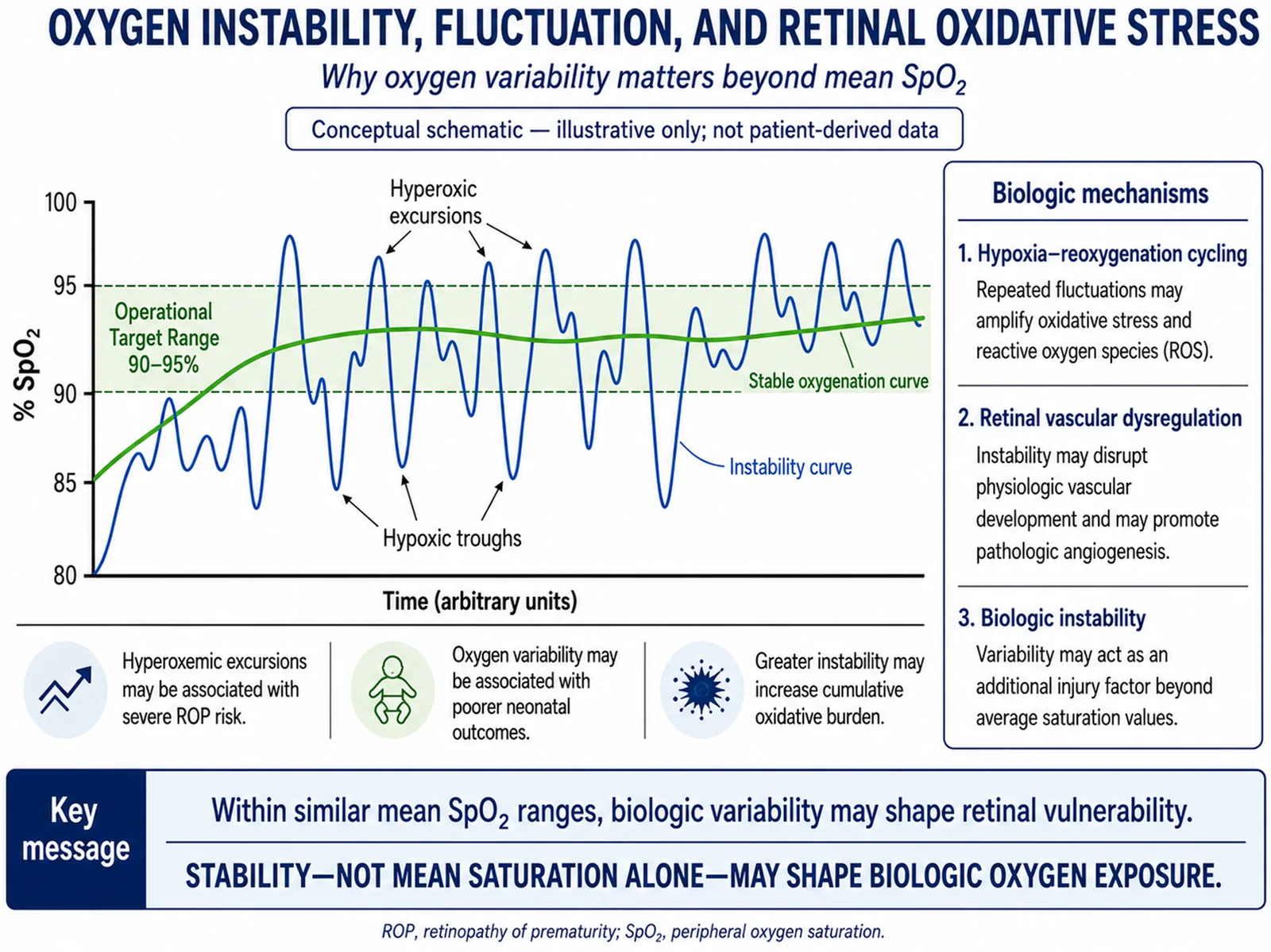
Conceptual illustration of oxygen instability, fluctuation, and retinal oxidative stress. This figure illustrates possible patterns of relatively stable and unstable oxygenation over time. The SpO_2_ traces are illustrative only and do not represent patient-derived monitoring data. Recurrent hypoxemic troughs and hyperoxemic excursions may contribute to hypoxia-reoxygenation stress, oxidative injury, and retinal vascular dysregulation, although these relationships require prospective validation within the DOSE framework. The figure is labelled as “Conceptual schematic—not data-derived.”

The conceptual relationship between oxygen instability, fluctuation burden, and retinal oxidative stress is illustrated in Figure 2.

ROP should also be understood as a multifactorial neurovascular disease rather than an oxygen-only disorder. In addition to oxygen exposure, important contributors include severity of prematurity, low birth weight, impaired postnatal growth, IGF-1-related pathways, infection, systemic inflammation, anemia, transfusion exposure, respiratory morbidity, mechanical ventilation, nutritional status, and other neonatal morbidities (2). Oxygen instability may interact with these biological and clinical vulnerabilities, thereby modifying the immature infant's susceptibility to retinal vascular dysregulation. This broader perspective is important to avoid reducing ROP pathogenesis to oxygen exposure alone.

Implementation context may further influence oxygen stability and ROP prevention. In low- and middle-income countries (LMICs), neonatal units may face practical challenges such as limited availability of blended oxygen, variable pulse oximeter quality, inconsistent probe positioning or signal reliability, alarm fatigue, high nurse-to-patient ratios, absence of standardized oxygen titration protocols, limited continuous monitoring, delayed ophthalmologic screening, referral barriers, and variable access to timely treatment (11–14). These system-level factors should not be interpreted as the sole explanation for differences in ROP outcomes, but they may interact with infant-level vulnerability and clinical care processes to amplify oxygen instability and delay prevention.

Although oxygen variability, intermittent hypoxemia, time in target range, and cumulative oxygen exposure have been discussed in the neonatal literature, they are often considered separately rather than integrated into a unified model of biologic oxygen exposure. This article therefore proposes the Dynamic Oxygen Stability and Exposure (DOSE) framework as a conceptual and hypothesis-generating model for dynamic oxygen stewardship in ROP prevention. DOSE does not replace existing SpO_2_ guidelines and is not a validated clinical score or predictive model. Rather, it aims to organize delivered oxygen input, physiologic SpO_2_ response, oxygen stability, and cumulative exposure into a structured framework that can guide future measurement, quality improvement, and prospective validation.

## 2 Rationale for the DOSE framework

Current oxygen management in preterm infants is largely organized around target SpO_2_ ranges. This approach is clinically necessary because bedside oxygen titration requires practical and measurable targets, and major oxygen-targeting trials have shaped contemporary practice by balancing mortality, systemic morbidity, and severe ROP outcomes across different saturation ranges (5–7). However, target ranges provide only a partial description of oxygen exposure. They indicate where saturation should ideally remain, but they do not fully describe how oxygenation fluctuates, how long an infant remains outside the target range, how much FiO_2_ is required to maintain a given SpO_2_, or how cumulative exposure evolves over time.

This distinction is important because oxygen exposure in neonatal intensive care is inherently dynamic. Preterm infants may experience repeated episodes of intermittent hypoxemia, hyperoxemic excursions, apnea-related desaturation, bradycardia-associated instability, respiratory support changes, probe-related artifacts, alarm delays, and frequent manual FiO_2_ adjustments. Studies in extremely preterm infants have shown that intermittent hypoxemia is common and may be influenced by oxygen-targeting strategies, while recurrent intermittent hypoxemia or bradycardia has been associated with adverse later outcomes (9, 10). As a result, two infants with comparable average SpO_2_ values may have substantially different oxygen exposure profiles. One infant may remain relatively stable within the target range, whereas another may experience recurrent oscillations between hypoxemia and hyperoxemia. These differences may be clinically meaningful even when conventional summary values appear similar.

Several existing concepts already capture parts of this problem, including intermittent hypoxemia, oxygen variability, time in target range, time outside target range, cumulative hyperoxic burden, cumulative hypoxic burden, and automated oxygen control. Automated oxygen-control studies and reviews have further emphasized the importance of maintaining infants within target range and reducing oxygen instability, although these approaches are not universally available and require appropriate clinical validation and implementation (15, 16). However, these concepts are often discussed separately. In clinical practice, oxygen management may therefore remain focused on achieving a target range rather than understanding the broader biologic pattern of oxygen exposure. A framework that integrates these dimensions may help clinicians and researchers conceptualize oxygen therapy not only as a target-based intervention, but also as a time-dependent physiologic exposure. This perspective is also consistent with previous Indonesian data showing that the use and control of supplemental oxygen were major risk factors for both the development and progression of ROP in preterm infants, supporting the importance of oxygen stewardship beyond target selection alone (17).

### 2.1 Biological mechanisms linking oxygen fluctuation to angiogenesis

Oxygen fluctuation may influence retinal vascular development through interconnected oxygen-sensitive pathways. During normal fetal development, oxygen availability is not static; rather, fetal oxygenation changes across gestation and may contribute to developmental signaling involved in vascular maturation (18–20). In preterm infants, premature transition from the relatively hypoxemic intrauterine environment to postnatal oxygen exposure may disrupt this developmental balance. Recurrent hypoxemia, hyperoxemic excursions, and hypoxia–reoxygenation cycles may generate oxidative stress, alter mitochondrial redox balance, activate inflammatory pathways, and impair endothelial function, thereby creating conditions that may influence retinal vascular development (2, 4).

At the molecular level, fluctuating oxygen exposure may affect oxygen-sensitive transcriptional pathways, including hypoxia-inducible factor signaling and downstream angiogenic mediators such as vascular endothelial growth factor. Recent evidence linking gestational oxygenation patterns to the coordinated expression of HIF1A, ADRB3, and VEGFA further supports the biological plausibility that fluctuations in oxygenation may interact with transcriptional programs involved in vascular development (21). However, DOSE should not be interpreted as suggesting that reduction of oxygen fluctuation alone is sufficient to prevent pathological vascularization. Rather, oxygen instability is best viewed as one potentially modifiable exposure that interacts with prematurity severity, postnatal growth, inflammation, transfusion exposure, respiratory morbidity, nutrition, and health-system factors.

Taken together, these biological considerations provide a mechanistic rationale for integrating oxygen dose, physiologic response, stability, and cumulative exposure into a unified conceptual framework.

## 3 Definition of DOSE components

The DOSE framework consists of four interconnected domains: D—Dose, O—Output, S—Stability, and E—Exposure. These domains are intended to describe oxygen therapy as a dynamic physiologic exposure rather than as a static saturation target alone. The framework does not propose new validated thresholds; instead, it provides a structured vocabulary for organizing oxygen-related variables that may be relevant to ROP vulnerability and future prospective validation. This approach builds on prior evidence showing that oxygen saturation targets, oxygen variability, intermittent hypoxemia, and cumulative exposure may influence neonatal outcomes and retinal vulnerability (4, 5, 7, 9, 10).

**Figure 3:**
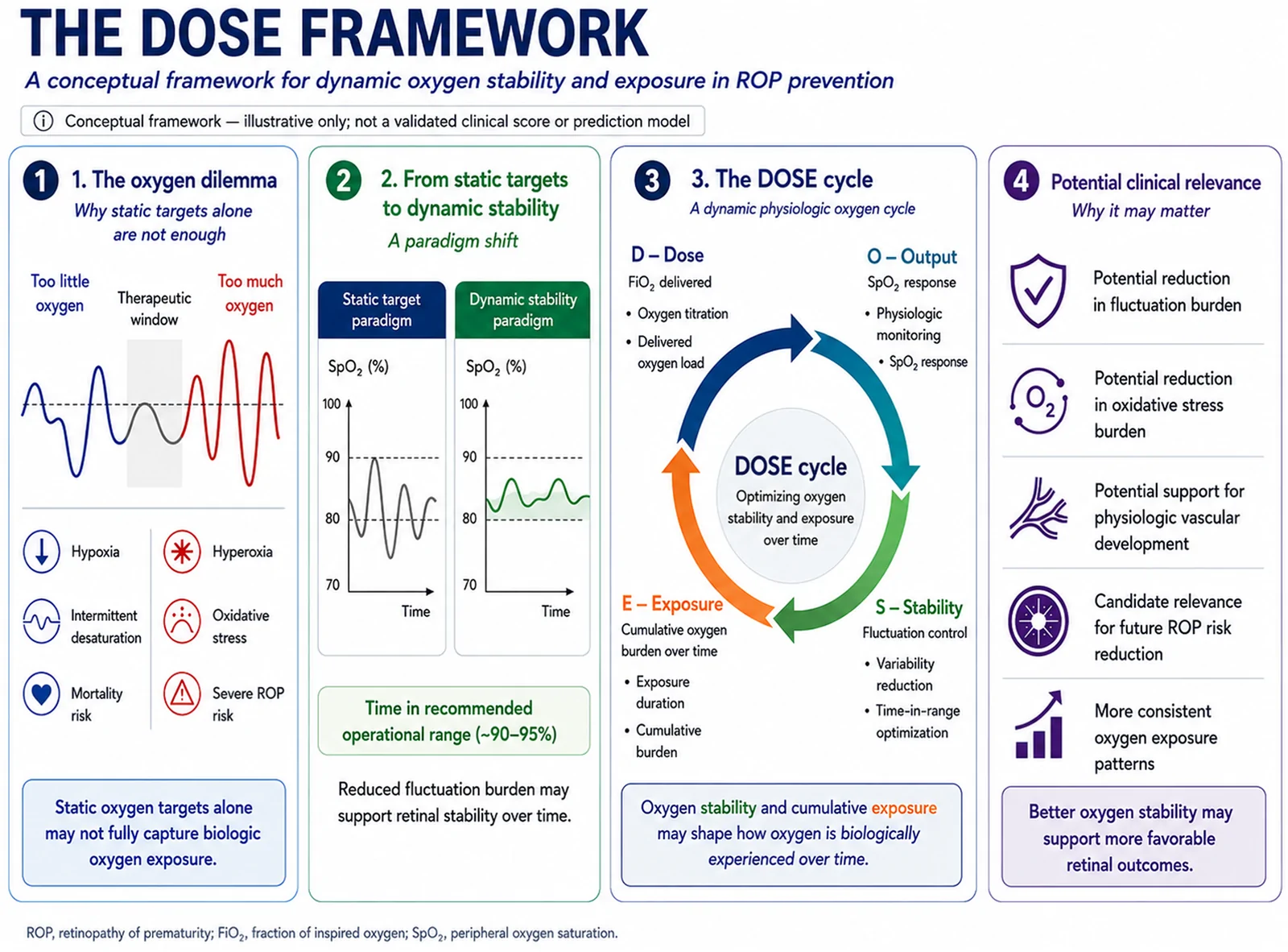
The DOSE framework: dynamic oxygen stability and exposure. This conceptual schematic presents DOSE as a framework integrating delivered oxygen input, physiologic oxygenation output, oxygen stability, and cumulative exposure over time. The figure is intended to organize dynamic oxygen-stewardship domains and should not be interpreted as a validated risk score or prediction model.

The four-domain structure of the DOSE framework is summarized in Figure 3.

### 3.1 D—dose: delivered oxygen input

In the DOSE framework, Dose refers to the oxygen delivered to the infant. Although FiO_2_ is the most commonly used bedside indicator of oxygen administration, FiO_2_ alone does not fully represent biologic oxygen exposure. The effective oxygen dose may also be influenced by duration of oxygen therapy, oxygen flow, respiratory support modality, lung disease severity, ventilation status, hemoglobin concentration, cardiac output, perfusion, and tissue oxygen delivery. This broader interpretation is consistent with the clinical complexity of oxygen therapy in preterm infants, in whom oxygen exposure reflects both delivered oxygen concentration and the infant's respiratory and circulatory condition (3, 4).

Therefore, “oxygen dose” in DOSE should be interpreted as a broader concept of delivered oxygen load rather than FiO_2_ alone. Candidate parameters may include mean FiO_2_, maximum FiO_2_, duration of oxygen therapy, time-weighted FiO_2_, cumulative FiO_2_-hours, and oxygen exposure stratified by respiratory support modality. This domain asks: how much oxygen support is delivered over time?

### 3.2 O—output: physiologic oxygenation response

Output refers to the infant's physiologic response to the delivery of oxygen, most commonly reflected by peripheral oxygen saturation measured by pulse oximetry. SpO_2_ is not simply a passive result of FiO_2_; it reflects the interaction between delivered oxygen, lung function, ventilation, circulation, hemoglobin concentration, oxygen affinity, perfusion, and signal quality. Large oxygen-targeting trials have relied on SpO_2_ as the practical bedside measure of oxygenation, but these trials also showed that target ranges alone do not eliminate differences in mortality, systemic morbidity, and severe ROP outcomes (5–7).

Within DOSE, Output is intended to capture the infant's response to oxygen input. Candidate parameters may include mean SpO_2_, median SpO_2_, time in target range, time below target range, time above target range, and the magnitude or speed of SpO_2_ response after FiO_2_ adjustment. This domain asks: how does the infant physiologically respond to the delivery of oxygen?

### 3.3 S—stability: fluctuation control

Stability refers to the consistency of oxygenation over time. It reflects the degree to which SpO_2_ remains within the intended target range and avoids recurrent hypoxemic or hyperoxemic excursions. Stability is central to the DOSE framework because infants with similar mean SpO_2_ values may have very different fluctuation patterns and, therefore, potentially different biological oxygen experiences. Prior studies have shown that lower SpO_2_ targets may be associated with increased intermittent hypoxemia, and that intermittent hypoxemia or bradycardia in extremely preterm infants may be associated with adverse later outcomes (9, 10).

Candidate parameters for stability may include SpO_2_ standard deviation, coefficient of variation, frequency of hypoxemic and hyperoxemic episodes, duration of desaturation or hyperoxemia, fluctuation amplitude, time outside the target range, frequency of FiO_2_ adjustments, and alarm burden. Automated oxygen-control research further supports the clinical relevance of reducing time outside target range and improving oxygen stability, although these approaches require appropriate validation and contextual adaptation (15, 16). These metrics remain candidates for future validation and should not be interpreted as established clinical thresholds. This domain asks: How stable is the infant's oxygenation over time?

### 3.4 E—exposure: cumulative oxygen burden over time

Exposure refers to the cumulative burden of oxygen-related physiologic experience over clinically relevant time windows. This domain integrates both the intensity and duration of oxygen exposure, including the cumulative time spent above or below defined saturation thresholds. It recognizes that oxygen-related vulnerability may depend not only on moment-to-moment values but also on the accumulated burden of fluctuation, hypoxemia, hyperoxemia, and oxygen requirement over time. This concept is biologically plausible because repeated hypoxia–reoxygenation cycles and hyperoxic exposure may contribute to oxidative stress, vascular dysregulation, and abnormal retinal angiogenesis (2, 4).

Candidate parameters may include cumulative hyperoxic and hypoxic burdens, cumulative time outside the target range, area above or below predefined SpO_2_ thresholds, time-integrated oxygen exposure, and longitudinal oxygen exposure profiles over the first days or weeks of life. This domain asks: What cumulative oxygen burden has the infant experienced over time?

Taken together, these four domains provide a structured way to describe oxygen exposure dynamically: Dose describes what is delivered, Output describes the physiologic response, Stability describes how consistently oxygenation is maintained, and Exposure describes the accumulated burden over time. The DOSE framework is therefore intended to support future research and quality improvement by moving beyond isolated SpO_2_ targets toward integrated assessment of oxygen input, response, variability, and cumulative exposure.

## 4 What is New in DOSE?

The novelty of DOSE does not lie in claiming that FiO_2_ exposure, SpO_2_ response, oxygen variability, intermittent hypoxemia, time in target range, or cumulative oxygen burden are individually new concepts. These domains have already been described in neonatal oxygen literature, including studies on oxygen saturation targets, intermittent hypoxemia, automated oxygen control, and oxygen-related retinal vulnerability (4, 5, 7, 9, 10, 16). Rather, DOSE provides a unified conceptual and translational framework that integrates these domains into a dynamic model of biologic oxygen exposure relevant to ROP vulnerability.

Existing oxygen-management strategies remain clinically essential because they define practical SpO_2_ target ranges for bedside care. Major oxygen-targeting trials and meta-analyses have shaped current practice by demonstrating the balance between mortality, systemic morbidity, and severe ROP across different saturation targets (5–7). However, target ranges alone do not fully describe how much oxygen is delivered, how the infant physiologically responds, how stable oxygenation remains, or how cumulative exposure develops over time. DOSE extends this target-based approach by organizing these dimensions into four linked domains: delivered oxygen input, physiologic oxygenation output, oxygen stability, and cumulative exposure.

Within this framework, FiO_2_ and SpO_2_ are interpreted as part of a dynamic exposure-response relationship rather than as isolated bedside values. A similar SpO_2_ value may represent different biologic oxygen experiences depending on the FiO_2_ required to achieve it, the duration of exposure, the frequency and amplitude of fluctuations, and the cumulative burden of hypoxemic or hyperoxemic events. This interpretation is consistent with evidence that intermittent hypoxemia and oxygen instability may be associated with adverse neonatal outcomes and may contribute biologically to oxidative stress and retinal vascular dysregulation (4, 9, 10). Thus, DOSE shifts attention from whether an infant is within a target range at a given moment toward how oxygen exposure is experienced across time.

DOSE should therefore be understood as a conceptual synthesis and translational oxygen-stewardship framework, not as a validated clinical score, predictive model, or replacement for existing oxygen-saturation guidelines. Its intended role is to provide a structured language for future research, quality improvement, and prospective validation of dynamic oxygen-exposure metrics.

This distinction is important for both high-resource and resource-limited settings. In high-resource neonatal units, DOSE-related metrics may be derived from continuous monitoring systems, automated oxygen-control platforms, or high-resolution physiologic data (15, 16). In low- and middle-income settings, simplified DOSE-informed approaches may support quality improvement by emphasizing oxygen blending, consistent titration, reduced time outside target range, alarm response, timely screening, referral, and treatment (14, 22). In both contexts, the aim is not to introduce a new oxygen target, but to improve understanding and stewardship of oxygen exposure as a dynamic and cumulative process.

## 5 Candidate metrics and testable hypotheses

For the DOSE framework to be evaluated empirically, each domain should be translated into candidate measurable parameters. These parameters are not proposed as validated clinical thresholds but rather as preliminary operational variables to support future research, quality improvement, and prospective validation. Their inclusion is intended to make DOSE more testable and to provide a structured roadmap for studying dynamic oxygen exposure in relation to ROP and other neonatal outcomes. Similar domains have been explored in the neonatal oxygen literature, including intermittent hypoxemia, oxygen variability, time in target range, automated oxygen control, and cumulative oxygen exposure, but these concepts have not yet been unified into a single DOSE-based operational framework for ROP prevention (9, 10, 15, 16). Table 1 summarizes candidate operational metrics for each DOSE domain and their future validation targets.

**Table 1:** Candidate operational metrics for the DOSE framework.

DOSE domain	Conceptual meaning	Candidate measurable parameters	Future validation target
D—Dose	Delivered oxygen input or oxygen load	Mean FiO_2_, maximum FiO_2_, duration of oxygen therapy, time-weighted FiO_2_, cumulative FiO_2_-hours, oxygen exposure by respiratory support modality	Association with ROP severity, treatment-requiring ROP, and oxygen-related morbidity
O—Output	Physiologic oxygenation response	Mean SpO_2_, median SpO_2_, time in target range, time below target range, time above target range, SpO_2_ response to FiO_2_ adjustment	Ability to distinguish stable from unstable oxygenation patterns and identify infants requiring closer monitoring
S—Stability	Fluctuation control	SpO_2_ standard deviation, coefficient of variation, frequency and duration of hypoxemic episodes, frequency and duration of hyperoxemic episodes, fluctuation amplitude, time outside target range, frequency of FiO_2_ adjustments, alarm burden	Prediction of ROP progression, systemic instability, and need for intensified oxygen stewardship
E—Exposure	Integrated oxygen burden over time	Cumulative hyperoxic burden, cumulative hypoxic burden, cumulative time outside target range, area above or below predefined SpO_2_ thresholds, time-integrated oxygen exposure	Dose-response relationship with ROP severity, treatment-requiring ROP, and longer-term outcomes

DOSE, Dynamic Oxygen Stability and Exposure; FiO_2_, fraction of inspired oxygen; SpO_2_, peripheral oxygen saturation; ROP, retinopathy of prematurity.

Several methodological issues should be standardized before these metrics can be compared across studies or implemented clinically. These include monitoring averaging time, sampling frequency, artefact removal, probe reliability, definitions of hypoxemia and hyperoxemia, selected SpO_2_ target ranges, exposure window duration, respiratory support status, and adjustment for infant-level risk factors. These issues are particularly important because intermittent hypoxemia and oxygen instability may be influenced by both infant physiology and monitoring practices, while automated oxygen-control studies also highlight the importance of measurement precision, alarm settings, and response algorithms (9, 15, 16). Without such standardization, DOSE-related metrics may vary substantially between units and may not be directly comparable.

The DOSE framework also enables the formulation of several testable hypotheses. First, among infants with similar mean SpO_2_ values, those with greater oxygen instability, reflected by higher SpO_2_ variability, more frequent hypoxemic or hyperoxemic episodes, or longer time outside the target range, may have a higher risk of severe or treatment-requiring ROP. This hypothesis is biologically plausible because recurrent hypoxia-reoxygenation cycles may contribute to oxidative stress, vascular dysregulation, and abnormal retinal angiogenesis (4). Second, cumulative hyperoxic and hypoxic burden may show stronger associations with ROP progression than mean SpO_2_ alone, particularly because major oxygen-targeting trials demonstrated that target ranges influence outcomes but do not fully explain inter-infant and inter-institutional variability (5–7). Third, infants requiring higher FiO_2_ to maintain similar SpO_2_ values may represent a distinct exposure-response phenotype with greater respiratory morbidity and potentially greater vulnerability to oxygen-related retinal injury.

Fourth, DOSE-related metrics may improve risk stratification when combined with established infant-level predictors such as gestational age, birth weight, postnatal growth, sepsis, transfusion exposure, anemia, nutritional status, and respiratory support. This is important because ROP is a multifactorial neurovascular disease rather than an oxygen-only disorder (2). Fifth, implementation of DOSE-informed quality-improvement strategies, such as improved oxygen blending, standardized titration protocols, alarm management, and reduction of time outside target range, may reduce oxygen instability and improve process measures related to ROP prevention, particularly when integrated with risk-based screening and prevention models (14, 23).

These hypotheses require prospective validation before clinical application. Future studies should evaluate whether DOSE-derived metrics are independently associated with ROP severity, treatment-requiring ROP, mortality, bronchopulmonary dysplasia, neurodevelopmental outcomes, and other oxygen-related morbidities. The framework should therefore be interpreted as a research and quality-improvement roadmap rather than as a completed risk-prediction model.

## 6 Integration with STOP–R1O2P3

The DOSE framework and the STOP–R1O2P3 continuum-of-care framework serve different but complementary purposes. DOSE is proposed as a biologic oxygen-exposure framework that organizes delivered oxygen input, physiologic oxygenation response, oxygen stability, and cumulative exposure over time. STOP–R1O2P3, in contrast, is an author-developed programmatic continuum-of-care framework for ROP prevention based on previous work in Indonesia (14). It should not be interpreted as an internationally validated guideline or predictive score, but as a practical implementation model for organizing ROP prevention across neonatal stabilization, oxygen stewardship, nutrition, infection prevention, ophthalmologic screening, referral, and timely treatment.

Within this structure, DOSE provides the physiologic rationale for dynamic oxygen stewardship, whereas STOP–R1O2P3 provides the systems-level pathway for embedding oxygen stewardship in clinical care. DOSE asks what should be understood and measured: how oxygen is delivered, how the infant responds, how stable oxygenation remains, and how exposure accumulates over time. STOP–R1O2P3 asks where and how these principles can be implemented across neonatal teams, nursing practice, referral systems, ophthalmology services, and broader health-system processes (14).

This distinction is important because oxygen instability is not only a physiologic bedside phenomenon but also a care-process and systems-level issue. Even when similar SpO_2_ targets are used, oxygen stability may be influenced by oxygen-blending capacity, pulse oximeter reliability, alarm settings, nurse-to-patient ratios, oxygen titration protocols, respiratory support capacity, monitoring continuity, and timeliness of clinical response. These implementation factors may affect how consistently oxygen targets are achieved and how rapidly hypoxemic or hyperoxemic excursions are recognized and corrected (14, 22).

In this integrated model, DOSE can be viewed as the biologic core of oxygen stewardship, while STOP–R1O2P3 provides the operational continuum for translating oxygen-stewardship principles into practice. The continuum includes antenatal and perinatal optimization, neonatal stabilization, oxygen monitoring, respiratory care, nutrition, infection prevention, risk-based ROP screening, referral coordination, ophthalmologic assessment, and timely treatment. These steps emphasize that ROP prevention depends not only on oxygen targeting, but also on coordinated multidisciplinary care involving neonatology, nursing, ophthalmology, nutrition, infection control, referral networks, and health-system coordination (14).

Therefore, integration of DOSE with STOP–R1O2P3 is intended to link oxygen physiology with implementation. DOSE helps define the dynamic oxygen-exposure domains that should be monitored, measured, and studied, whereas STOP–R1O2P3 helps define the care pathway through which these principles can be operationalized. Together, they provide a conceptual bridge between oxygen biology, bedside monitoring, quality improvement, and health-system coordination for ROP prevention.

**Figure 4:**
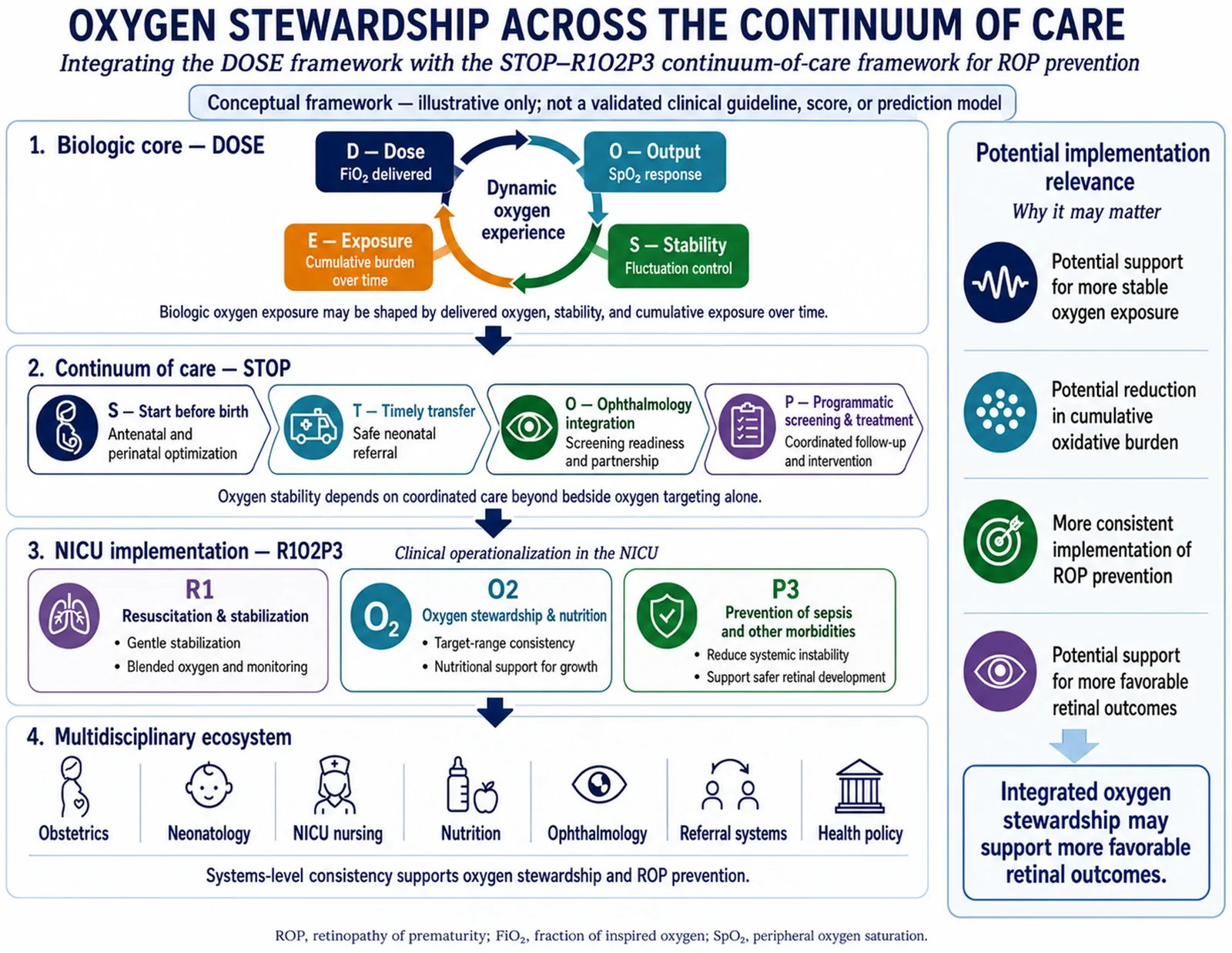
Oxygen stewardship across the continuum of care: integration of DOSE with STOP–R1O2P3. This conceptual schematic illustrates the complementary relationship between DOSE, a biologic oxygen-exposure framework, and STOP–R1O2P3, a systems-level continuum-of-care framework for ROP prevention. The figure is intended as an implementation aid and does not represent an internationally validated guideline or prediction model.

The conceptual integration of DOSE with STOP–R1O2P3 across the continuum of care is summarized in Figure 4.

## 7 Implementation considerations in LMIC settings

The DOSE framework may be particularly relevant in low- and middle-income countries (LMICs), where improvements in neonatal survival may not always be matched by equivalent access to oxygen-monitoring infrastructure, ophthalmologic screening, referral pathways, and timely treatment. The global burden of ROP-related visual impairment is concentrated substantially in middle-income regions, where neonatal survival has improved but prevention, screening, and treatment systems remain variable (24, 25). However, healthcare-system variability should not be interpreted as the sole explanation for differences in ROP outcomes. ROP remains multifactorial, and implementation challenges may interact with infant-level vulnerability, severity of prematurity, respiratory morbidity, sepsis, anemia, transfusion exposure, impaired postnatal growth, nutritional status, and screening or treatment access (2, 14).

In many LMIC neonatal units, oxygen instability may be amplified by practical bedside barriers. These include limited availability of blended oxygen, variable pulse oximeter quality, inconsistent probe positioning and signal reliability, limited access to continuous monitoring, alarm fatigue, high nurse-to-patient ratios, absence of standardized oxygen titration protocols, and variability in respiratory support capacity. Reviews of neonatal oxygen therapy in LMICs have noted gaps in oxygen monitoring, including facilities where oxygen may be delivered without adequate monitoring equipment, while limited access to oxygen blenders can increase the risk of imprecise oxygen delivery and hyperoxia in resource-limited settings (11–13). These factors may increase time outside target range, delay recognition of hypoxemic or hyperoxemic excursions, and make consistent oxygen titration more difficult.

The DOSE framework may help organize these challenges into measurable and modifiable domains. The Dose domain may be affected by limited oxygen blending, inconsistent FiO_2_ delivery, and variable respiratory support availability. The Output domain may be affected by pulse oximeter accuracy, probe placement, perfusion, motion artifact, and monitor averaging time. The Stability domain may be affected by alarm settings, alarm fatigue, staffing ratios, titration response time, apnea burden, and manual FiO_2_ adjustment practices. The Exposure domain may be affected by prolonged oxygen therapy, cumulative time outside target range, delayed escalation or weaning, and inconsistent documentation of oxygen exposure over time.

Implementation also extends beyond the bedside. ROP prevention requires reliable access to screening, timely ophthalmologic assessment, appropriate referral pathways, and the availability of treatment, such as laser or anti-VEGF therapy, when indicated. In middle-income and resource-limited settings, insufficient resources for examining and treating at-risk infants, delayed referral, and variable treatment availability have been recognized as key contributors to avoidable ROP-related blindness (14, 24, 25). Programmatic experience also suggests that oxygen stewardship should be linked with timely screening and treatment rather than implemented as an isolated bedside intervention (22).

A DOSE-informed implementation strategy in LMIC settings does not necessarily require advanced automated oxygen-control systems at the outset. Initial quality-improvement steps may include ensuring access to oxygen blenders when possible, standardizing SpO_2_ target ranges and alarm limits, improving pulse oximeter probe placement and signal reliability, training nurses and physicians in oxygen titration protocols, reducing avoidable time outside target range, auditing episodes of hypoxemia and hyperoxemia, strengthening referral pathways, and linking oxygen stewardship with ROP screening and treatment programs.

Therefore, in LMIC settings, the value of DOSE lies not only in future high-resolution physiologic monitoring but also in providing a practical structure for identifying where oxygen instability occurs and where quality-improvement interventions can be targeted. By connecting bedside oxygen delivery with monitoring reliability, staffing, protocols, screening access, referral, and treatment availability, DOSE may support a more integrated approach to ROP prevention across diverse healthcare systems.

## 8 Limitations

Several limitations should be acknowledged. First, the DOSE framework is currently a conceptual and hypothesis-generating model. It has not yet been prospectively validated as a clinical score, risk-prediction model, or decision-support tool. Therefore, DOSE should not be used to guide individual clinical decisions or to replace existing oxygen saturation guidelines. Current SpO_2_ target ranges remain essential operational standards for neonatal oxygen management and are supported by major oxygen-targeting trials and meta-analyses (5–7).

Second, the proposed DOSE metrics remain candidate operational parameters rather than validated thresholds. Measures such as time-weighted FiO_2_, time in target range, SpO_2_ standard deviation, coefficient of variation, frequency of hypoxemic or hyperoxemic episodes, cumulative hyperoxic burden, cumulative hypoxic burden, and area above or below predefined thresholds require prospective evaluation before they can be interpreted clinically. Their predictive value, optimal cut-off points, exposure windows, and relationship with ROP severity remain uncertain.

Third, DOSE-related measurements may be affected by technical and methodological variability. Pulse oximeter averaging time, sampling frequency, probe placement, motion artefact, perfusion status, alarm settings, monitor brand, definitions of hypoxemia and hyperoxemia, respiratory support modality, and documentation quality may all influence measured oxygen exposure. Without standardization, DOSE metrics may not be directly comparable across neonatal units, datasets, or healthcare systems.

Fourth, the framework focuses on oxygen stewardship and should not be interpreted as a complete model of ROP pathogenesis. ROP is a multifactorial neurovascular disease influenced by gestational age, birth weight, severity of prematurity, impaired postnatal growth, IGF-1-related pathways, sepsis, systemic inflammation, anemia, transfusion exposure, respiratory morbidity, mechanical ventilation, nutrition, and other neonatal morbidities (2). Oxygen instability may represent one potentially modifiable contributor within this broader network, but it should not be considered the sole determinant of retinal vascular disease.

Fifth, the relevance and feasibility of DOSE implementation may differ across healthcare settings. High-resource neonatal units may be able to derive DOSE-related metrics from continuous monitoring systems, automated oxygen-control platforms, or high-resolution physiologic data (15, 16). In contrast, many LMIC settings may require simplified quality-improvement approaches focused on oxygen blending, pulse oximeter reliability, titration protocols, staffing, screening access, referral systems, and treatment availability (11, 12, 14).

Finally, because this article does not analyze original patient-level data, it cannot establish causal relationships between DOSE-related metrics and ROP outcomes. The framework is intended to generate testable hypotheses and guide future prospective studies. Multicenter validation will be required to determine whether DOSE-derived metrics add predictive value beyond established ROP risk factors and whether DOSE-informed quality-improvement strategies can reduce oxygen instability, severe ROP, treatment-requiring ROP, or other oxygen-related neonatal morbidities.

## 9 Future directions

Future research should move from conceptual development toward prospective validation of DOSE-related metrics. Studies should evaluate whether dynamic oxygen-exposure variables, including time-weighted FiO_2_, time in target range, SpO_2_ variability, intermittent hypoxemia, hyperoxemic excursions, cumulative hypoxic burden, cumulative hyperoxic burden, and area above or below predefined thresholds, are independently associated with ROP severity and treatment-requiring ROP. These analyses should adjust for established ROP risk factors, including gestational age, birth weight, postnatal growth, sepsis, transfusion exposure, respiratory morbidity, nutrition, and other neonatal morbidities (2, 9, 10).

Multicenter prospective studies are needed to determine whether DOSE-derived metrics add predictive value beyond conventional oxygen targets and established clinical risk factors. Such studies should include diverse neonatal populations, different levels of respiratory support, and varied healthcare settings. Importantly, future work should compare infants with similar mean SpO_2_ values but different patterns of oxygen instability to determine whether fluctuation burden and cumulative exposure provide additional prognostic information beyond average saturation alone (5–7).

High-resolution continuous monitoring data may provide an important foundation for DOSE validation. Continuous SpO_2_ and FiO_2_ recordings could allow detailed characterization of oxygen exposure profiles, including fluctuation frequency, duration of hypoxemic and hyperoxemic episodes, response to FiO_2_ adjustment, and cumulative time outside target range. Automated oxygen-control systems and precision monitoring platforms may also offer opportunities to study whether improved oxygen stability translates into better retinal, respiratory, and systemic outcomes (15, 16).

Future studies should also evaluate whether DOSE metrics are associated with outcomes beyond ROP. Intermittent hypoxemia and oxygen instability have been linked with adverse neonatal and neurodevelopmental outcomes, suggesting that dynamic oxygen exposure may have systemic relevance beyond retinal vascular disease (10). Therefore, validation studies should consider treatment-requiring ROP, bronchopulmonary dysplasia, mortality, neurodevelopmental impairment, and other oxygen-related morbidities as clinically important endpoints.

In LMIC settings, future research should also assess simplified DOSE-informed quality-improvement strategies. These may include improved access to oxygen blenders, standardized SpO_2_ targets and alarm limits, pulse oximeter quality improvement, staff training in oxygen titration, reduction of time outside target range, structured referral pathways, and integration of oxygen stewardship with ROP screening and timely treatment. Such implementation studies are particularly important where neonatal survival is improving but monitoring, staffing, referral, and treatment resources remain variable (11, 12, 14, 22).

Ultimately, future validation should determine whether DOSE is useful as a research measurement framework, a quality-improvement tool, or a basis for future predictive modeling. Until such validation is available, DOSE should be interpreted as a conceptual and hypothesis-generating framework designed to stimulate more precise study of dynamic oxygen exposure in ROP prevention.

## 10 Conclusion

ROP prevention requires attention not only to oxygen saturation targets, but also to the dynamic pattern of oxygen exposure experienced by preterm infants over time. Current SpO_2_ target ranges remain essential for neonatal practice and should not be replaced. However, target ranges alone may not fully capture the burden of fluctuations, oxygen instability, cumulative exposure, or differences in biologic oxygen experience among infants with similar average saturation values.

The DOSE framework is proposed as a conceptual and hypothesis-generating model for dynamic oxygen stewardship in ROP prevention. By organizing delivered oxygen input, physiologic oxygenation response, oxygen stability, and cumulative exposure into a unified structure, DOSE may provide a useful language for future research, quality improvement, and prospective validation. It should not be interpreted as a validated clinical score, predictive model, or substitute for existing oxygen-management guidelines.

Importantly, DOSE should be considered within the broader multifactorial context of ROP. Oxygen instability may represent one potentially modifiable contributor, but retinal vulnerability is also shaped by prematurity severity, postnatal growth, inflammation, anemia, transfusion exposure, respiratory morbidity, nutrition, screening access, referral systems, and treatment availability. Integration with continuum-of-care approaches such as STOP–R1O2P3 may help connect oxygen physiology with practical implementation across neonatal and ophthalmologic care pathways.

Future studies are needed to determine whether DOSE-derived metrics improve prediction of ROP severity, treatment-requiring ROP, systemic morbidity, or neurodevelopmental outcomes beyond established clinical risk factors. Until such validation is available, DOSE should be viewed as a structured conceptual framework to guide more precise investigation of dynamic oxygen exposure and to support safer, more consistent oxygen stewardship in diverse neonatal settings.

## Data Availability

The original contributions presented in the study are included in the article/supplementary material, further inquiries can be directed to the corresponding author.
